# Fluacrypyrim Protects Hematopoietic Stem and Progenitor Cells against Irradiation via Apoptosis Prevention

**DOI:** 10.3390/molecules29040816

**Published:** 2024-02-09

**Authors:** Xuewen Zhang, Zizhi Qiao, Bo Guan, Fangming Wang, Xing Shen, Hui Shu, Yajun Shan, Yuwen Cong, Shuang Xing, Zuyin Yu

**Affiliations:** 1 Beijing Key Laboratory for Radiobiology, Department of Experimental Hematology and Biochemistry, Beijing Institute of Radiation Medicine, Beijing 100850, China; 2 The Affiliated Suzhou Hospital of Nanjing Medical University, Suzhou Municipal Hospital, Gusu School, Nanjing Medical University, Suzhou 215000, China; 3 School of Life Science, Anhui Medical University, Hefei 230032, China

**Keywords:** ionizing radiation, hematopoietic stem cells, Fluacrypyrim, apoptosis, p53-PUMA signaling

## Abstract

Ionizing radiation (IR)-induced hematopoietic injury has become a global concern in the past decade. The underlying cause of this condition is a compromised hematopoietic reserve, and this kind of hematopoietic injury could result in infection or bleeding, in addition to lethal mishaps. Therefore, developing an effective treatment for this condition is imperative. Fluacrypyrim (FAPM) is a recognized effective inhibitor of STAT3, which exhibits anti-inflammation and anti-tumor effects in hematopoietic disorders. In this context, the present study aimed to determine whether FAPM could serve as a curative agent in hematopoietic-acute radiation syndrome (H-ARS) after total body irradiation (TBI). The results revealed that the peritoneally injection of FAPM could effectively promote mice survival after lethal dose irradiation. In addition, promising recovery of peripheral blood, bone marrow (BM) cell counts, hematopoietic stem cell (HSC) cellularity, BM colony-forming ability, and HSC reconstituting ability upon FAPM treatment after sublethal dose irradiation was noted. Furthermore, FAPM could reduce IR-induced apoptosis in hematopoietic stem and progenitor cells (HSPCs) both in vitro and in vivo. Specifically, FAPM could downregulate the expressions of p53-PUMA pathway target genes, such as Puma, Bax, and Noxa. These results suggested that FAPM played a protective role in IR-induced hematopoietic damage and that the possible underlying mechanism was the modulation of apoptotic activities in HSCs.

## 1. Introduction

Injury to human tissues due to irradiation was reported first in the year 1945 during the unfortunate event of the Hiroshima and Nagasaki atomic bomb explosion [1]. It is concerning that the rapid development of nuclear technology and its expanding application in the fields of medicine, industry, and military have only extended the casualty of mishaps due to irradiation to thousands of personnel [2,3]. The hematopoietic system in the human body is the most vulnerable to irradiation, and a hematopoietic injury could surface even at irradiation of less than 1 Gy, with the symptoms of diminished blood cell count conditions, such as anemia, thrombocytopenia, and particularly leukopenia [4,5]. The increasing scenarios of tension among the armed forces of different nations and terrorist threats, combined with the widespread application of radiation in oncology, have rendered it almost impossible to prevent radiation exposure, which underscores the prevalence of hematopoietic injury and the associated death rates.

Currently, the treatment of hematopoietic injury focuses mainly on the in-time replenishment of various blood cells, such as erythrocytes, platelet transfusion, hematopoietic stem cell (HSC) transplantation, and application of molecularly cloned hematopoietic growth factors [6,7]. Cell transplant presents a rather unsatisfactory benefit-to-risk ratio of cells, and the growth factors lead to appreciable outcomes only when the stem cells are maintained in a sound state, which emphasizes the necessity of ensuring the quantity and quality of HSCs [8,9]. In addition, for long-term physical recovery, it is essential to maintain the viability of HSCs [10]. Currently, only four cytokine-based medical countermeasures, including Filgrastim, Neulastat, Sargramostim, and Romiplostim, have been approved by the Food and Drug Administration (FDA) as radiomitigators to improve survival in patients received a myelosuppressive dose of radiation [11]. However, no radioprotector has received approval from FDA; it is imperative to explore and develop novel drugs for HSC protection and restoration after ionizing radiation (IR) [12].

FAPM is a synthetic β-methoxyacrylates compound, which has been identified as a potent inhibitor of STAT3 activation and exhibits salutary effects when used in the treatment of myeloid leukemia [13,14]. Although all kinds of myeloid injury share basic disorders such as inflammation and oxidative stress, it remains to be elucidated whether FAPM would be beneficial in the treatment of IR-induced myeloid injury. In this context, the present study aimed to evaluate, for the first time, the potential effects of FAPM on hematopoietic system injury after IR. The results revealed that FAPM could mitigate IR-induced hematopoietic system injury mainly by preventing apoptosis in the HSCs, both in vitro and in vivo.

## 2. Results

### 2.1. FAPM Administration Could Benefit the Hematological Recovery after Irradiation

**Figure 1:**
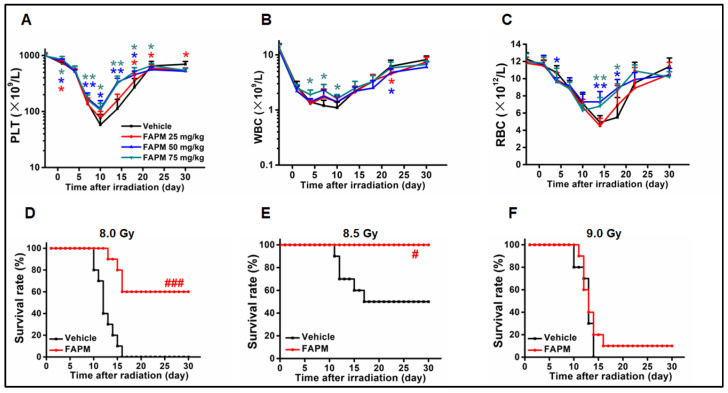
FAPM ameliorated pancytopenia and improved survival in the mice subjected to irradiation. (**A**–**C**) The mice were administered with vehicle or FAPM at different doses and then exposed to 6.5 Gy TBI. The (**A**) platelets, (**B**) white blood cells, and (**C**) red blood cells were analyzed in peripheral blood (*n* = 6). (**D**–**F**) Survival rates of the mice administrated with vehicle or FAPM followed by exposure to 8.0, 8.5, or 9.0 Gy TBI (*n* = 10). Data represent the mean ± standard deviation. * *p* < 0.05 and ** *p* < 0.01 versus the vehicle group by two-tailed Student’s *t*-test. # *p* < 0.05 and ### *p* < 0.001 versus the vehicle group by log-rank test.

In order to determine whether FAPM could accelerate the hematopoietic recovery after irradiation, vehicle or FAPM was administered at different doses to mice followed by subjecting the mice to sublethal irradiation (6.5 Gy). Afterward, the peripheral blood cell counts were measured at different time points. It was revealed that the administration of FAPM resulted in better peripheral blood (PB) cell counts, particularly PLT and RBC (Figure 1A–C), compared to the vehicle group, during the 10 to 14 days after irradiation. These results implied that FAPM could ameliorate pancytopenia in the mice subjected to irradiation, and among all three dosages, the 50 mg/kg dosage of FAPM performed the best and was, therefore, used in the subsequent experiments.

### 2.2. FAPM Improved the Survival Rate of Mice after Lethal Irradiation

To assess whether FAPM conferred radioprotection to the mice subjected to lethal irradiation, the mice were injected with the vehicle or FAPM (50 mg/kg) followed by irradiation as described earlier. Three doses (8.0 Gy, 8.5 Gy, and 9.0 Gy) of irradiation were applied, and in two low-dose exposure groups, improvement was noted. The FAPM exhibited excellent improvement effects (Figure 1D,E) in the 8.0 Gy and 8.5 Gy irradiation groups (10 of 10 mice and 6 of 10 mice, respectively). However, the protective effects of FAPM were largely compromised (1 of 10 mice only) when the mice were subjected to 9.0 Gy (Figure 1F). These results indicated that FAPM could exert apparent effects on the survival rate of mice subjected to different degrees of irradiation ranging from 8.0 Gy to 8.5 Gy.

### 2.3. FAPM Alleviated Irradiation-Induced Injury to BM

**Figure 2:**
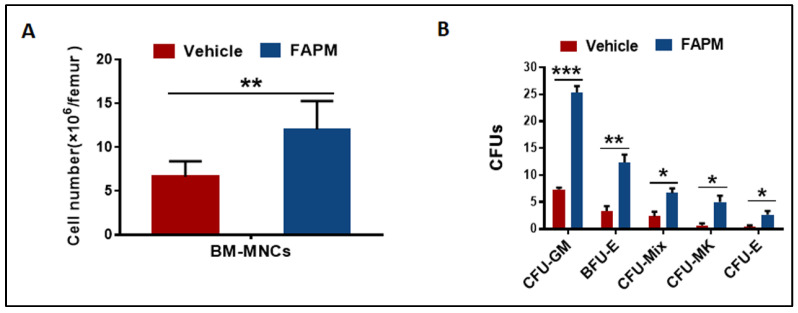
FAPM alleviated the irradiation-induced injury in BM and enhanced colony-forming ability in the mice subjected to irradiation. The vehicle and FAPM group mice were administered vehicle and FAPM, respectively, followed by exposure to 6.5 Gy TBI (*n* = 7). (**A**) Bone marrow mononuclear cell (BMNC) counts and (**B**) colony-forming units were determined for the mice treated with the vehicle or FAPM at 10 days after exposure to 6.5 Gy TBI. Data represent the mean ± standard deviation. * *p* < 0.05, ** *p* < 0.01, and *** *p* < 0.001 versus the vehicle group by two-tailed Student’s *t*-test.

To further investigate the protective effect of FAPM on murine bone marrow, BMNCs were collected and evaluated 10 days after the 6.5 Gy TBI. The results revealed that compared to the vehicle group, the FAPM administration group exhibited a nearly two-fold elevation in the BMNC counts (Figure 2A). In addition, the CFU assay revealed marked improvement in the BM-derived clonogenic activity, particularly in the granulocyte-macrophage CFUs (CFU-GM), erythroid burst-forming units (BFU-E), and CFU-MIX, in the FAPM-treated mice compared to the mice administered with only vehicle (Figure 2B).

### 2.4. FAPM Accelerated the Recovery of HSPCs after Irradiation Exposure

**Figure 3:**
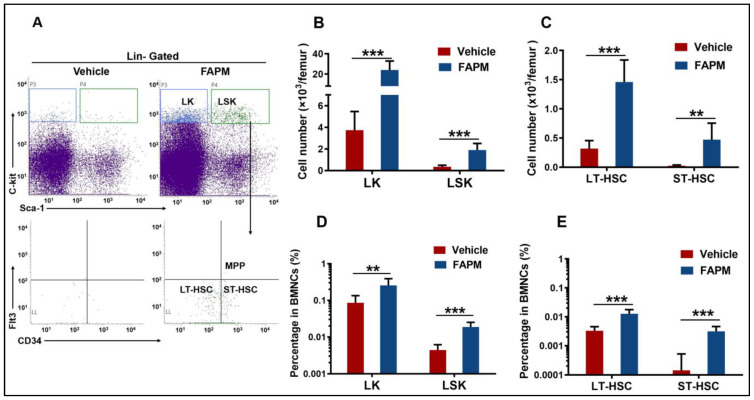
FAPM accelerated hematopoietic stem and progenitor cell recovery in the mice subjected to irradiation. The vehicle and FAPM group mice were administered vehicle and FAPM, respectively, followed by exposure to 6.5 Gy TBI (*n* = 7). (**A**) Representative gating strategy of LT-HSC, ST-HSC, and MPP was analyzed by flow cytometry. Bone marrow cells were harvested 10 days after the irradiation and evaluated using flow cytometry. (**B**,**C**) Absolute numbers and (**D**,**E**) and percentages of LK (Lin–Sca1–c-Kit+) cells, LSK (Lin–Sca1+c-Kit+) cells, long-term hematopoietic stem cells (LT-HSC) (LSK Flt3– CD34–), short-term HSCs (ST-HSCs) (LSK Flt3–CD34+), and multipotent progenitor (MPP) (LSK Flt3+ CD34+) cells in the bone marrow nuclear cells (BMNCs). Data represent the mean ± standard deviation. ** *p* < 0.01 and *** *p* < 0.001 versus the vehicle group by two-tailed Student’s *t*-test.

To further compare the effect of FAPM in promoting the recovery of HSPCs after irradiation, mice were administered vehicle or FAPM followed by exposure to 6.5 Gy TBI and then the flow cytometric analysis to investigate the dynamics of the BM hematopoietic stem cells (LSK) and progenitor cells (LK) 10 days after TBI. The absolute number (Figure 3B) and frequency (Figure 3D) of the LSK cells and LK cells were significantly increased in the FAPM-treated mice compared to the vehicle-treated mice. In addition, three cellular subpopulations of cells were identified, namely, long-term HSCs (LT-HSCs), short-term HSCs (ST-HSCs), and multipotent progenitor cells (MPPs). In the vehicle and FAPM-treated groups, MPP was nearly undetectable, while FAPM treatment led to 3-fold and 17-fold increases in the absolute numbers of LT-HSCs and ST-HSCs, respectively, compared to those in the vehicle-treated mice (Figure 3C). Consistent with this, 4-fold and 22-fold increases were noted in the frequency of LT-HSC and ST-HSC, respectively, in the BMNCs treated with FAPM compared to those treated with the vehicle, after TBI (Figure 3E). These results demonstrated that FAPM administration contributed to the recovery of HSPCs and is, therefore, promising for the peripheral blood cell recovery after irradiation.

### 2.5. FAPM Enhanced the Self-Renewal Capacity of HSCs after Irradiation

**Figure 4:**
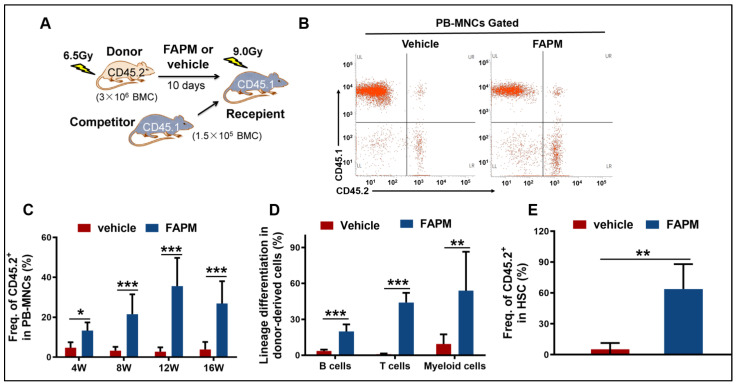
FAPM enhanced the repopulating ability of murine hematopoietic stem cells (HSCs), as revealed in the competitive repopulation experiment. (**A**) Schematic of the experimental procedure. (**B**) Representative FACS plots. (**C**) The percentage of the donor (CD45.2) chimeras in the peripheral blood of the recipient mice (CD45.1) in the vehicle and FAPM groups at 4, 8, 12, and 16 weeks after transplantation (*n* = 7). (**D**) Frequency of vehicle-derived and FAPM donor (CD45.2)-derived T (CD45.2+CD3+) lymphocytes, B(CD45.2+B220+) lymphocytes, and myeloid cells (CD45.2+CD11b+ Gr-1+) in the peripheral blood of the recipient mice (CD45.1) at 12 weeks after the transplantation. (**E**) Percentages of LSK cells among the donor (CD45.2)-derived lymphocytes when the sacrifice was performed 16 weeks after the transplantation. Data represent the mean ± standard deviation. * *p* < 0.05, ** *p* < 0.01, and *** *p* < 0.001 versus the vehicle group by two-tailed Student’s *t*-test.

Considering the effect of FAPM in terms of accelerating HSPC recovery, competitive repopulation experiments were performed next to evaluate the self-renewal capacity of HSCs in the mice subjected to irradiation. The results revealed an evident increase in the CD45.2+ chimeras upon FAPM administration at different time points after the transplantation of peripheral blood mono-nuclear cells (PB-MNCs) (Figure 4C). Furthermore, the influence of HSCs on other blood cell lineages was investigated at 12 weeks after transplantation, and promising results were obtained in the FAPM administration group, in terms of the differentiation of mature T cells, B cells, and myeloid cells, compared to the vehicle administration group (Figure 4D). Regarding the frequency of donor-derived LSK cells in the recipient irradiated BM cells, the FAPM-treated group exhibited ten-fold higher values compared to the recipient irradiated control LSK cells (Figure 4E). These results demonstrated that FAPM could, in addition to improving the self-renewal capacity of irradiated HSCs, accelerate the multi-lineage reconstitution of peripheral blood after BM transplantation.

### 2.6. FAPM Mitigated Cell Apoptosis Both In Vitro and In Vivo

**Figure 5:**
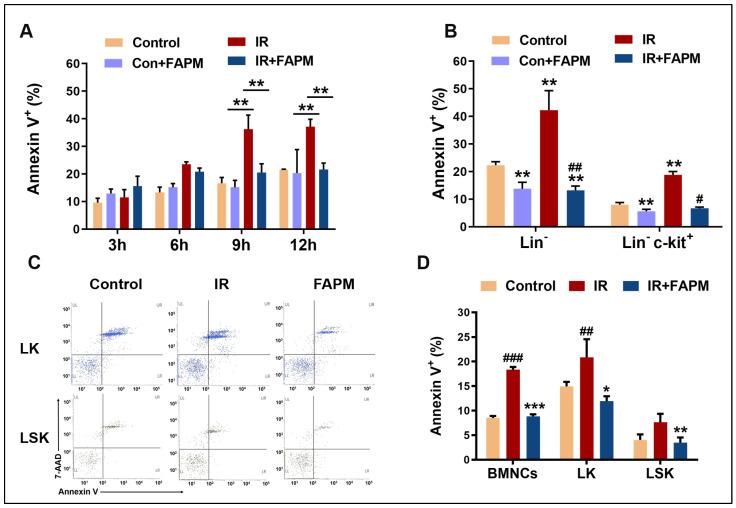
FAPM alleviated the irradiation-induced cell apoptosis both in vitro and in vivo. (**A**) The ratio of apoptosis in BMNCs at 3, 6, 9, and 12 h after the 6.5 Gy irradiation (*n* = 3). (**B**) The ratio of apoptosis in the Lin– cells and Lin–c-kit+ cells at 9 h after the 6.5 Gy irradiation. (**C**) Representative FACS plots. (**D**) Percentage of Annexin V ^+^ cells in BMNC, LK, and LSK cells at 6 h after 6.5 Gy TBI (*n* = 3). * *p* < 0.05, ** *p* < 0.01, and *** *p* < 0.001 versus the IR group by two-tailed Student’s *t*-test; # *p* < 0.05, ## *p* < 0.01, and ### *p* < 0.001 versus the control group by two-tailed Student’s *t*-test.

To determine whether the effect of FAPM in terms of hematopoietic recovery involves protection against HSC apoptosis after irradiation, a flow cytometric analysis was performed to evaluate HSPC apoptosis both in vitro and in vivo. In the in vitro experiments, after 9 h and 12 h of 6.5 Gy irradiation, the apoptosis rate of BM cells in the FAPM-treated group was significantly lowered compared to the IR group, while no difference was observed between the two groups in the early stage (3 and 6 h after irradiation) (Figure 5A). Next, the apoptotic rates of Lin– and Lin–c-Kit+ cells were determined at 9 h after 6.5 Gy of irradiation, and approximately 70% reduction in the apoptotic rate was observed in the FAPM-treated group compared to the control group (Figure 5B). In the in vivo experiments, IR caused different degrees of damage to BMNCs, LK cells, and LSK cells, and FAPM treatment led to 50% recovery in all three kinds of cells (Figure 5D).

### 2.7. FAPM Decreased Cell Apoptosis by Regulating the p53-PUMA Pathway

**Figure 6:**
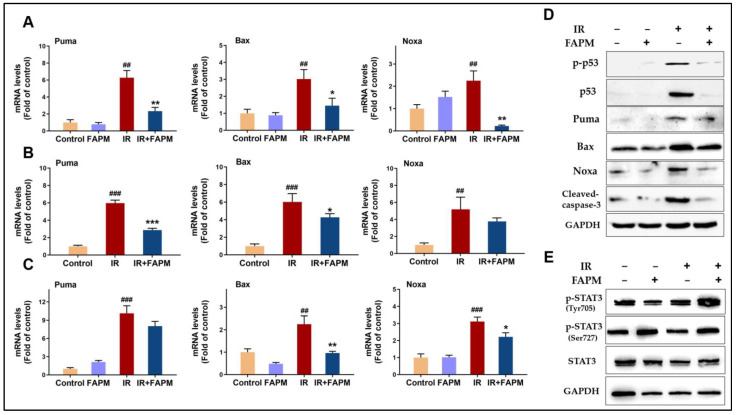
FAPM inhibited the irradiation-induced expressions of the genes related to the p53-PUMA pathway, both in vitro and in vivo. (**A**) Mice were sacrificed to collect and prepare the BMNCs, which were then pre-incubated with FAPM (5 µM) for 1 h followed by exposure to 6.5 Gy γ-irradiation and culture for another 9 h to obtain the total RNA. (**B**) The IR and FAPM groups were administered with the vehicle and FAPM, respectively, followed by exposure to 6.5 Gy TBI (*n* = 3). Control mice were administered with the vehicle and shielded from irradiation. The mice were sacrificed at 6 h after the irradiation to harvest BMNCs, from which the total RNA was obtained. (**C**) HSPCs in these BMNCs were enriched using magnetic-activated cell sorting (MACS) and then incubated with FAPM (5 µM) for 1 h, followed by exposure to 6.5 Gy γ-irradiation and culture for another 9 h to obtain the total RNA. RT-qPCR was performed to determine the mRNA expressions of Puma, Bax, and Noxa. (**D**,**E**) After incubation with FAPM (5 μM) for 1 h, BMNC cells were exposed to γ-irradiation at a dose of 6.5 Gy and cultured for another 3 h, and the BMNCs were harvested. The cell lysates were subjected to SDS-PAGE followed by Western blotting with p-p53, p53, Puma, Bax, Noxa, cleaved caspase-3, phospho-STAT3 (Tyr705), phospho-STAT3 (Ser727), STAT3, and GAPDH antibodies. Data represent the mean ± standard deviation. * *p* < 0.05, ** *p* < 0.01, and *** *p* < 0.001 versus IR group by two-tailed Student’s *t*-test; ## *p* < 0.01 and ### *p* < 0.001 versus control group by two-tailed Student’s *t*-test.

It is well recognized that the p53-PUMA pathway is strongly associated with the apoptosis induced by radiation in HSPCs, and a loss of p53 or PUMA confers resistance to irradiation by blocking apoptosis [15,16]. In this context, the mRNA expressions of the genes related to the p53-PUMA pathway were evaluated in the BMNCs in vitro. The results revealed that the expression levels of Puma, Bax, and Noxa were markedly downregulated in the FAPM-treated group (Figure 6A), and the same trend repeated in the in vivo experiments (Figure 6B). In addition, HSPCs were collected and incubated in vitro with FAPM, and the subsequent analysis revealed the same trend after FAPM treatment (Figure 6C). Consistent with the mRNA data, the expression level of protein of p-p53, p53, Puma, Bax, Noxa, and cleaved Caspase-3 (Figure 6D) was significant downregulate on the FAPM-treated group, which were involved in the regulation of cell apoptosis. Although FAPM acts as a STAT3 activation inhibitor when used in the treatment of human myeloid leukemia cell lines in our previous study [13]. FAPM shows an activating effect on both STAT3 Tyr705 phosphorylation and STAT3 Ser727 phosphorylation when treated in BMNCs in combination with irradiation but has no significant effect on total STAT3 protein. (Figure 6E). Collectively, these results suggested that the anti-apoptotic effect of FAPM on hematopoietic cells in the mice subjected to IR might be exerted through the suppression of the p53-PUMA pathway.

## 3. Discussion

Protection against radiation exposure remains a challenge for military personnel and the potentially afflicted citizens, particularly the damage caused to the hematopoietic system as it is the most susceptible to radiation-induced damage [1,15,17]. Several countermeasures to radiation exposure are available for mitigating the hematopoietic injury after TBI [17,18,19]. However, the medical arsenal is relatively limited as only Filgrastim, Neulastat, Sargramostim, and Romiplostim [11] have been officially approved by the FDA as medical approaches for treating hematopoietic-acute radiation syndrome (H-ARS). In addition, the transportation, preservation/storage, and production costs of these measures are high. Therefore, effective strategies to prevent or treat hematopoietic injury are lacking currently.

FAPM is a strobilurin derivative with a β-methoxyacrylate structure, which was applied as an agricultural bactericide and an inhibitor of the mitochondrial cytochrome bc1 complex [20]. According to a previous study, FAMP exhibits antinociceptive and anti-inflammatory effects [14,21]. The previous studies conducted by our research group on FAPM revealed its antitumorigenic effects on the hematopoietic system [13,22], and this study is, to the best of our knowledge, the first to report the potential of FAPM in terms of protection against radiation exposure. The feasibility, cost-friendliness, and great clinical prospect of FAPM renders it potentially beneficial as a pharmaceutical agent providing protection against irradiation.

IR might induce severe oxidative stress in the hematopoietic system, which could damage the DNA, proteins, and lipids in HSCs, all of which would culminate in cell death [23]. Subjects exposed to IR undergo hematopoietic system myelosuppression and present with neutropenia and thrombocytopenia that is eventually fatal. Consistent with this, this study revealed that the intraperitoneal administration of FAPM could greatly promote the survival of mice subjected to 8.0 Gy and 8.5 Gy of TBI, although a further increase in the irradiation dose to 9.0 Gy resulted in the protective effects of FAPM to be largely compromised. HSPC injury and the subsequent BM failure are the primary causes of death following irradiation exposure [24]. In this study, FAPM treatment significantly increased the percentage and the absolute number of HSPCs in the BM of the mice subjected to IR, enhanced the whole cell counts of BM, and led to better performance in terms of CFUs. It was demonstrated that FAPM could reserve the cellular count of multi-lineages of peripheral blood cells after 6.5 Gy TBI, which indicated a lower probability of bleeding, infection, and tissue oxygenation. It is noteworthy that TBI could, in addition to causing acute BM injury, lead to long-term BM suppression, both of which manifested as cellular aging and exhaustion of the HSC reserve [1,24,25,26,27], and the pathological lesions of which share several similarities in terms of pathophysiological progression, particularly the involvement of irradiation-induced oxidative stress and senescence [28,29,30]. Notably, FAPM could effectively improve myelosuppression, as revealed in the competitive reconstitution experiments, which indicated that FAPM could maintain the functioning of HSCs in the mice subjected to irradiation, resulting in improved hematopoietic regenerative capacity and better multi-lineage recovery. Considering the satisfactory performance of FAPM in terms of HSPC protection at an early stage after irradiation, it is also of significance to investigate the long-term protective potential of FAPM against TBI.

It is well recognized that the p53 gene and the associated signaling pathway play important roles in the regulation of apoptosis. Previous studies have confirmed that targeting pertinent molecules in the p53 pathway enables efficiently promoting the recovery of IR-induced BM injury [15,25,31]. Therefore, whether the p53-associated signaling pathway was involved in the protective effect of FAPM against IR was investigated next in this study. According to the flow cytometry results, FAPM exhibited significant anti-apoptosis effects on the BM cells after irradiation, both in vitro and in vivo, particularly its effect of reserving the counts of certain LK cells and LSK cells. Further, the expressions of anti- and pro-apoptosis molecules associated with the p53-PUMA pathway were evaluated using qPCR and Western blot. The results revealed that FAPM could effectively downregulate the expression of puma, Bax, and Noxa at the mRNA levels. As for protein levels, the expression of p-p53, p53, Puma, Bax, Noxa, and cleaved Caspase-3 were significant suppressed after FAPM treatment, all of which are signature pro-apoptosis molecules. This demonstration of the effect of FAPM on the p53-PUMA pathway would serve as strong physiological evidence in favor of the anti-radiative property of FAPM, thereby laying a solid foundation for the promising clinical research and development potential of FAPM. Since our studies have shown that the radioprotection of FAPM is strongly associated with the p53-PUMA pathway, while mice constitutively lacking alleles of the p53 gene were highly susceptible to developing lymphomas and sarcomas [32]. Therefore, it is essential to observe that whether FAPM treatment promotes oncogenesis in the long term, as well as FAPM treatment combined with irradiation. It has been reported that an increase in STAT3 activity correlates with inhibition of p53 expression [33]. Several studies have proven that constitutively activated of STAT3 confers radioprotection in irradiated mice [34,35]. Our results presented that FAPM promote the activation of STAT3 when they are treated in BMNCs; however, whether STAT3 mediates the role of FAPM in p53- PUMA pathway remains unknown. In summary, further experimental studies are required to determine whether STAT3 regulates FAPM on hematopoietic cells in mice exposed to IR by inhibiting the p53-PUMA pathway.

According to the findings of the present study and the other ones stated above, it may be postulated that the protective effects of FAPM on HSCs against apoptosis occur under the regulation of multiple pathways operating in a coordinated manner. Therefore, future studies must conduct, further experiments using auxiliary scientific approaches, such as RNA sequencing, to obtain insights into the precise mechanism underlying the protective effects of FAPM against irradiation.

## 4. Materials and Methods

### 4.1. Reagents

The FAPM used in the present study was synthesized as described in a previous report [13]. The flow cytometry antibodies CD117(c-kit), Flt3, CD34, CD45.1, CD45.2, CD3, Lineage (CD3/Gr-1/CD11b/CD45R(B220), Ly6G/Ly-6c(Gr-1), CD45R/B220, and Annxin V were purchased from Biolegend (San Diego, CA, USA). Ly-6A/E(Sca-1), CD11b, and 7-Amino-Actinomycin D (7-AAD) were obtained from BD eBiosciences (San Diego, CA, USA). MethoCult GF 3434 StemSpan media and Red Blood Cell Lysis Buffer were from StemCell Technologies (Vancouver, BC, Canada). RPMI1640 medium, fetal bovine serum (FBS), and phosphate-buffered saline (PBS) were supplied by Gibco (New York, NY, USA).

### 4.2. Mice

The animals used in the present study were male C57BL/6J (CD45.2) mice and male B6.SJL/BoyJ (CD45.1) mice, which were procured from Beijing HFK Bioscience (Beijing, China) and Beijing Vital River Laboratory Animal Technology (Beijing, China), respectively. Experimental animals were 6 to 8 weeks old and were kept in the standard environment, which was a room temperature of 22 ± 2 °C and 12 h of light during the day, as well as pellet food and water at liberty. When we sacrificed the mice, we adopted the method of cervical dislocation, and intraperitoneally injection of sodium pentobarbital was applied for anesthesia when necessary. All procedures used in the present study were reviewed and approved by the Animal Care and Use Committee of Animal Center in Academy (IACUC-DWZX-2022-836).

### 4.3. Irradiation and FAPM Administration

The mice were subjected to either a sublethal dose (6.5 Gy) or lethal dose (8.0, 8.5, or 9.0 Gy) of total body irradiation (TBI) using a ^60^Co γ-ray source at a dose rate of approximately 62 cGy/min. The mice had been intraperitoneally injected with FAPM at different doses or the empty vehicle (phosphate-buffered saline, PBS) at 48 h, 24 h, and 3 h prior to irradiation.

### 4.4. Peripheral Blood Cell Counts

The peripheral blood cell counts were monitored based on the calculations described in a previous report [11]. To examine the most appropriate dosage of administration, C57BL/J was given FAPM (20, 50, or 75 mg/kg) or vehicle at 48, 24, and 3 h before irradiation, and then subjected to a sublethal dose of TBI, which allowed a fully restoration of peripheral blood (PB) cells within four weeks. Twenty-four mice were randomly apported to 4 groups and given vehicle or FAPM before TBI, after which the PB cell counts were recorded on a weekly basis as previously presented [36].

### 4.5. BM Cellularity and BM-Derived Clonogenic Activity Assay

Bone marrow nuclear cells (BMNCs) for the BM count assay were collected by flushing the femurs with 1 mL of RPMI-1640 medium. The collected single-cell suspension was then evaluated as described in a previous report [37]. In addition, a BM-derived clonogenic activity assay was performed, in which the BM nuclear cells were cultured for colony-forming units in MethoCult medium, followed by the identification and quantification of the developed colonies 7 days after the procedure, using the protocol described in a previous report [37].

### 4.6. Mouse Competitive Repopulation Assay

We first prepared two groups of B6.SJL mice (CD45.1) mice as our recipients and put them under 9 Gy of irradiation for hematopoietic system damage. Then, 3 × 10^6^ cells from the donor mice (CD45.2) which were given FAPM or vehicle 10 days after 6.5 Gy irradiation were retrieved and mixed with 1.5 × 10^5^ cells from the competitor mice (CD45.1); the doner cell sets were injected to the recipients accordingly. Lastly, we tested the chimeric rates of CD45.1^+^ and CD45.2^+^ cells of the peripheral blood in number by cytometry analysis at 4-, 8-, 12-, and 16-weeks post transplantation.

### 4.7. Flow Cytometry Analysis

Briefly, the BMNCs were collected by flushing the femur and tibia of mice, and the red blood cells were lysed using the lysis buffer. For hematopoietic cell phenotypic analysis, 1 × 10^7^ BM cells were incubated with antibodies, and then the cells were washed with PBS, after that, the frequencies of LK cells, LSK cells, short-term HSCs, multipotent progenitor cells, and long-term HSCs were analyzed with a flow cytometer (Thermo Fisher Scientific, Waltham, MA, USA). For HSPC apoptosis in vivo, mice were given either vehicle or FAPM, and then exposed to a sublethal dose (6.5 Gy) of TBI. Mice were sacrificed at 6 h after irradiation; BM cells were first stained with HSPC markers. Next, cells were incubated with Annexin V and 7-AAD. For HSPC apoptosis in vitro, we collect BM cells first, and then use magnetic bead enrichment to obtain Lin^-^ and Lin^-^c-kit^+^ cells, following the manufacturer’s instructions. After that, divide each cell population into the following four groups: (A) Control, (B) Control + FAPM, (C) IR, and (D) IR + FAPM. The IR and IR + FAPM groups were exposed to 6.5 Gy radiation; the Control + FAPM and IR + FAPM groups were cultured in RPMI-1640 medium containing 10% fetal bovine serum with FAPM (5 μmol/L) for 1 h before 6.5 Gy irradiation. At 9 h post irradiation, cells were collected and incubated with Annexin V and PI for flow cytometer analysis.

### 4.8. Quantitative Real-Time Polymerase Chain Reaction (qRT-PCR)

Total RNA was isolated from the BMNCs using the Trizol reagent (Invitrogen, Carlsbad, CA, USA). Then, cDNA was synthesized using Reverse Transcriptase kit (Takara, Kusatsu, Japan). The primers were synthesized by Tianyi Biotech (Beijing, China). Real-time quantitative PCR was performed with UltraSYBR One Step RT-qPCR Kit (CWBIO, Taizhou, China) on the CFX96 TouchTM Real-Time PCR Detection system (Bio-Rad, Hercules, CA, USA). The primers are used as follows: β-actin (forward 5′-AGAGGGAAATCGTGCGTGAC-3′ and reverse 5′- CAATAGTGATGACCTGGCCGT-3′), Puma (forward 5′-ACCGCTCCACCTGCCGTCA-3′ and reverse 5′-ACGGGCGACTCTAAGTGCT-3′), Noxa (forward 5′-GGAGTGCACCGGACATAACT-3′ and reverse 5′-TTGAGCACACTCGTCCTTCA-3′), and Bax (forward 5′-TTGCTGATGGCAACTT CAAC-3′ and reverse 5′-GATCAGCTCGGGCACTTTAG-3′). The threshold cycle (CT) values for each reaction were determined and averaged using TaqMan SDS analysis software (Applied Biosystems 2.1, Foster City, CA, USA). Changes in gene expression were calculated by the comparative CT method (fold changes = 2^−ΔΔCT^).

### 4.9. Western Blotting

At the end of the desired treatment, total cell lysates preparation and Western blot analyses were performed according to the procedure described before [38]. Puma (B-6, sc-377015) and Noxa (114c307) were purchased from Santa Cruz Biotechnology (Santa Cruz, CA, USA); Bax, p53 (1c12), phospho-p53 (Ser15), cleaved Caspase-3 (ASP175), Phospho-STAT3 (Tyr705), Phospho-STAT3 (Ser727), and STAT3 (124H6) were supplied by Cell Signaling Technology (Danvers, MA, USA).

### 4.10. Statistical Analysis

Two-tailed Student’s *t*-test was used for analytical comparisons between two groups. Survival was evaluated using Kaplan–Meier analysis along with the Log-Rank test. *p*-value of <0.05 was considered statistically significant. All figures were drawn by using GraphPad Prism 8.0 (San Diego, CA, USA).

## 5. Conclusions

This is the first demonstration that FAPM exhibits protective efficacy against IR-induced hematopoietic injury. Prophylactic FAPM administration could significantly improve survival and peripheral blood cell recovery in irradiated mice. Consistently, FAPM-treated, irradiated mice showed obvious increases in the counts of BM mononuclear cells (BMNC) and BM-derived CFUs. Moreover, FAPM administration markedly promoted HPSC regeneration and HSC repopulation in the BM of irradiated mice. Mechanistically, in vivo and in vitro FAPM administration could ameliorate cell apoptosis in BMNC and BM-HPSC from irradiated mice, which may be attributed to transcriptional inhibitory effects of FAPM on downstream genes of p53-PUMA pathway.

## Data Availability

Research data are stored in an institutional repository and will be shared upon request to the corresponding author.
